# An Efficient Modular Gateway Recombinase-Based Gene Stacking System for Generating Multi-Trait Transgenic Plants

**DOI:** 10.3390/plants11040488

**Published:** 2022-02-11

**Authors:** Guannan Qin, Suting Wu, Liying Zhang, Yanyao Li, Chunmei Liu, Jianghui Yu, Lihua Deng, Guoying Xiao, Zhiguo Zhang

**Affiliations:** 1 Key Laboratory of Agro-Ecological Processes in Subtropical Region, Institute of Subtropical Agriculture, Chinese Academy of Sciences, Changsha 410125, China; qinguannan13@mails.ucas.edu.cn (G.Q.); liyanyao19@mails.ucas.ac.cn (Y.L.); liuchunmei15@mails.ucas.edu.cn (C.L.); yujianghui@isa.ac.cn (J.Y.); denglihua@isa.ac.cn (L.D.); 2 Biotechnology Research Institute, Chinese Academy of Agricultural Sciences, Beijing 100081, China; 82101201609@caas.cn (S.W.); 82101211023@caas.cn (L.Z.); 3 University of Chinese Academy of Sciences, Beijing 100049, China; 4 Hainan Yazhou Bay Seed Laboratory, Sanya 572025, China

**Keywords:** multi-transgene stacking, gateway recombination, golden gate cloning, modular, plants

## Abstract

Transgenic technology can transfer favorable traits regardless of reproductive isolation and is an important method in plant synthetic biology and genetic improvement. Complex metabolic pathway modification and pyramiding breeding strategies often require the introduction of multiple genes at once, but the current vector assembly systems for constructing multigene expression cassettes are not completely satisfactory. In this study, a new in vitro gene stacking system, GuanNan Stacking (GNS), was developed. Through the introduction of Type IIS restriction enzyme-mediated Golden Gate cloning, GNS allows the modular, standardized assembly of target gene expression cassettes. Because of the introduction of Gateway recombination, GNS facilitates the cloning of superlarge transgene expression cassettes, allows multiple expression cassettes to be efficiently assembled in a binary vector simultaneously, and is compatible with the Cre enzyme-mediated marker deletion mechanism. The linked dual positive-negative marker selection strategy ensures the efficient acquisition of target recombinant plasmids without prokaryotic selection markers in the T-DNA region. The host-independent negative selection marker combined with the TAC backbone ensures the cloning and transfer of large T-DNAs (>100 kb). Using the GNS system, we constructed a binary vector containing five foreign gene expression cassettes and obtained transgenic rice carrying the target traits, proving that the method developed in this research is a powerful tool for plant metabolic engineering and compound trait transgenic breeding.

## 1. Introduction

In contrast to traditional breeding techniques such as mutagenesis and hybridization, transgenic technology can accurately transfer advantageous traits to recipient plants regardless of reproductive isolation. Transgene stacking is the method of combining two or more foreign genes in the same plant. Genetically modified crop varieties with stacked traits can meet wider and more complex user needs and show more commercial value than single-trait varieties [1].

Gene stacking can be achieved by methods such as hybridization, cotransformation, retransformation, or the transformation of a single vector expressing multiple genes [2,3,4]. Single-vector transformation with multigene expression cassettes (ECs) presents advantages of timesaving and cosegregation, so it is particularly favored by researchers [5,6]. However, some technical obstacles to the implementation of this strategy remain, such as the construction, propagation, and plant transformation of large vectors carrying multiple foreign genes. A number of strategies for realizing the construction of multigene stacking vectors have been reported. For example, the MultiRound Gateway system [7], GANNTRY system [8] and TGSII system [9] use site-specific recombinases. The jStack system [10] utilizes the endogenous homologous recombination machinery of yeast. Goderis et al. [11] reported a modular transgene stacking system based on homing endonucleases. The GoldenBraid [12] approach uses Type IIS restriction enzymes iteratively to assemble standardized modules. However, a transgene stacking system combining the advantages of a simple principle, modularity and a high positive rate is still an important requirement for plant synthetic biology.

Gateway technology is a universal cloning method based on bacteriophage lambda (λ) recombination at specific sequences referred to as *att* sites [13]. The intergradation of λ into the *Escherichia coli* chromosome (i.e., the lysogenic pathway or BP reaction) was characterized by the transformation of *att*B and *att*P sites into *att*L and *att*R sites, catalyzed by the protein combination of λ Int (Integrase) and *E. coli* IHF (Intergradation Host Factor) [14]. The commercial BP enzyme, which is a combination of λ Int and *E. coli* IHF, facilitates the in vitro simulation of natural BP reactions to obtain recombinant DNA molecules. Similarly, the lytic pathway, or LR reaction, has been simulated in vitro by using a commercial LR enzyme mix to transform *att*L and *att*R sites into *att*B and *att*P sites. By introducing different mutant *att* sites, a multisite LR reaction can further assemble up to four DNA fragments simultaneously [15].

Golden Gate (GG) assembly involves the application of a Type IIS restriction enzyme and a DNA ligase [16]. Type IIS restriction enzymes can cut double-stranded DNA outside of their recognition sites to form sticky ends. By setting the recognition site directions and overhang sequences, cleaved DNA fragments that do not contain recognition sites are obtained, and the overhang sequences are customized. In GG cloning, the Type IIS restriction enzyme and DNA ligase are placed in the same tube for reaction. The Type IIS restriction enzyme is responsible for producing sticky DNA without fragment recognition sites, and the DNA ligase is responsible for splicing these fragments together. The absence of recognition sites results in seamless cloning, and the spliced products are no longer recognized by the Type IIS restriction enzyme, thus enriching the target products. Customizable sticky ends allow the direction, number and order of the spliced DNA fragments to be controlled. Precloned common elements such as promoters with preset overhangs can be reused to achieve modularity, which is a key feature of synthetic biology [17].

In this study, we adopted a strategy similar to the MultiRound Gateway system [7] and introduced the large-capacity TAC backbone of the TGSII system [9] to develop a new gene stacking system, referred to as GuanNan Stacking (GNS). The GNS not only combines the advantages of Gateway recombination for efficient assembly of large fragments and GG cloning for modular assembly of small fragments, but also integrates the advantages of highly efficient positive selection and scarless negative selection, which makes the acquisition of expected recombinants simple and efficient. Using the GNS system, a proof-of-concept binary vector containing five gene expression cassettes, including two selectable markers and three reporter genes, was constructed and then was used to transform rice. The genotype and phenotype identification of regenerated plants verified the existence and expression of the transgenes, indicating the feasibility of the GNS system. Some commonly used promoters, coding sequences (CDSs), terminators and expression cassette modules were provided to facilitate the use of the GNS system by researchers.

## 2. Results

### 2.1. Principles and New Definitions of the GNS System

The GNS system consists of three types of plasmids: donor vectors, element modules and destination vectors.

**Figure 1:**
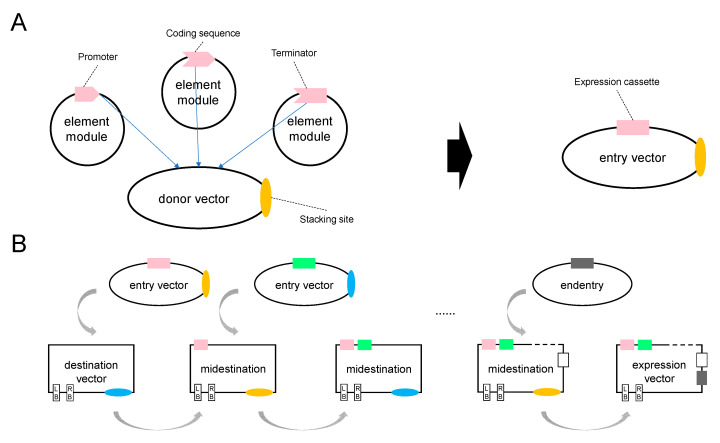
Principal illustration of the GNS system. (**A**) An entry vector assembled using the backbone of a donor vector and three element modules. Each element module carries a component of an expression cassette, i.e., promoter, CDS or terminator. Through freely selecting and combining different reusable element modules, diversified cargos in the form of expression cassettes meeting wide needs of users can be built modularly. Stacking sites on the entry vectors confer the ability to accept cargos on other entry vectors. (**B**) The stacking process uses a destination vector and entry vectors. A destination initially contains a stacking site for accepting the cargo on a compatible entry vector, at the same time, a new stacking site on the entry vector will also be integrated on the destination vector, generating a midestination. Entry vectors of adjacent rounds carry different and incompatible stacking sites. The stacking of cargos is accompanied by the abolishment of old stacking sites and regeneration of new ones. In the last step, an endentry, an entry vector without stacking sites, will be used to terminate the stacking process.

In the GNS system, elements that are commonly used in plant genetic engineering, including promoters, CDSs, terminators and expression cassettes, are provided in the form of precloned plasmids. These element modules can be reused and freely combined to assemble them into donor vector backbones, thus producing entry vectors in a modular manner (Figure 1A).

A destination vector is a binary vector used for *Agrobacterium*-mediated plant transformation and accepts target “CARGO” sequences carried by entry vectors. The binary vector obtained in each round of stacking is denoted as a MIDESTINATION. Due to the special design of the entry vectors, a midestination retains the ability to accept new cargos. To terminate the iterative stacking process, an entry vector that is incapable of restacking, denoted as ENDENTRY, is introduced in the last step to obtain the final expression vector (Figure 1B). General binary destination vectors with *att*R1 and *att*R2 recombination sites are compatible with the GNS system. The GNS system also provides two types of easy-to-use destination vectors based on pCAMBIA vector backbones and TAC backbones.

### 2.2. Assembly of Entry Vectors

The construction of entry vectors can be completed by either highly efficient GG assembly or BP recombination. The assembly of cargo using modular GG cloning needs to be specifically domesticated [18] to remove the recognition site of the Type IIS restriction endonuclease *Bsa*I via synonymous codon substitution. The assembly of cargo using the BP reaction is independent of sequence features, but only a single fragment can be assembled at a time, making this approach suitable for assembling single and undomesticatable DNA fragments, such as promoters whose *cis*-acting elements happen to contain *Bsa*I recognition sites.

**Figure 2:**
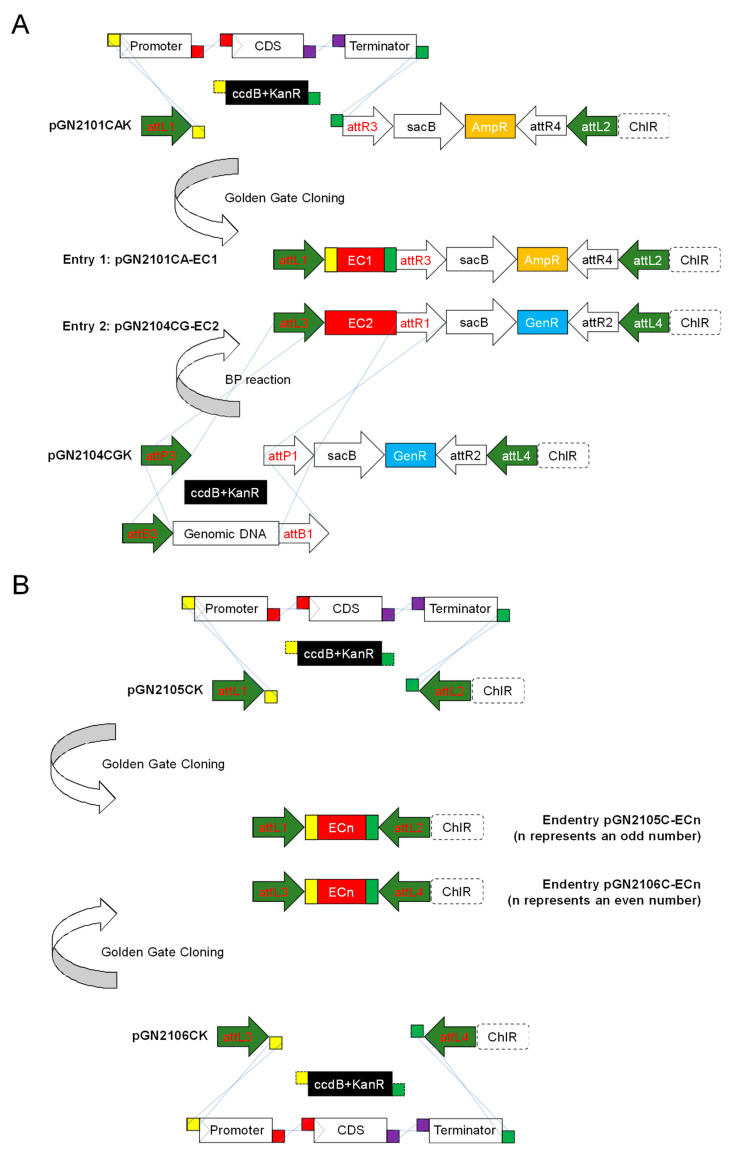
Assembly of entry vectors. (**A**) Assembly of entry vectors for intermediate stacking via GG cloning or BP recombination. Using pGN2101CAK as the donor vector, which is resistant to chloramphenicol, ampicillin and kanamycin, precloned inserts including promoter, CDS and terminator can be assembled to replace the lethal *ccdB* (linked with kanamycin resistance gene *KanR*) via GG cloning. The recombinant pGN2101CA-EC1 carries the first expression cassette (EC) and is resistant to chloramphenicol and ampicillin, conferred by *ChlR* and *AmpR*, respectively. Using pGN2104CGK as the donor vector, which is resistant to chloramphenicol, gentamicin and kanamycin, *att*B-flanked genomic DNA can be integrated to replace the lethal *ccdB* (linked with *KanR*) via BP recombination. The recombinant pGN2104CG-EC2 is resistant to chloramphenicol and gentamicin and carries the second EC. (**B**) GG assembly of endentries for the final stacking in odd or even round, respectively. Donor vectors pGN2105CK and pGN2106CK are resistant to chloramphenicol and kanamycin. Either pGN2105CK or pGN2106CK carries only one pair of *att*L sites flanking the lethal *ccdB* (linked with *KanR*); the *Bsa*I recognition sites adjacent inward the *att*L sites allow GG assembly of DNA fragments. Specifically, the combination of *att*L1/*att*L2 is located in pGN2105CK, while *att*L3/*att*L4 is located in pGN2106CK, and the corresponding endentry that loses kanamycin resistance is applied to terminating stacking process in odd and even rounds, respectively.

The minimal vector requirement for achieving the basic function of the GNS system includes six donor vectors and one destination vector (Table 1). In Figure 2, the basic GNS system is taken as an example to illustrate the typical assembly process of entry vectors.

All donor vectors are characterized by a pair of *att*L sites for LR recombination. To confer restacking ability, another pair of incompatible *att*R sites is placed inside the *att*L sites. To improve assembly efficiency, a common feature of the donor vectors is the linkage of the lethal gene *ccdB* [19] and the kanamycin resistance gene *KanR* outside of the *att*R combination as the target region to be replaced by the cargos. Inside the *att*L sites and adjacent to the target region, the sucrose-sensitive gene *sacB* [20] is linked to the ampicillin or gentamicin resistance gene (*AmpR* or *GenR*) and flanked by the pair of *att*R sites.

Specifically, the *att* sites of pGN2101CAK and pGN2103CAK are designed for odd-numbered round stacking and carry *AmpR* linked to *sacB*, while the *att* sites of pGN2102CGK and pGN2104CGK are designed for even-numbered round stacking and carry *GenR* linked to *sacB*. The assembly of entry vectors using the backbones of pGN2101CAK and pGN2102CGK can be performed via GG cloning. The assembly of entry vectors using the backbones of pGN2103CAK and pGN2104CGK requires the BP reaction (Figure 2A). The endentries require the use of the pGN2105CK or pGN2106CK backbone as well as GG cloning, which correspond to the final reactions in odd and even rounds, respectively (Figure 2B).

For the convenience of users, the names of all vectors referenced in this study reflect their resistance and are represented with letters after a number. In these identifiers, A represents ampicillin, C represents chloramphenicol, G represents gentamicin, and K represents kanamycin. Correspondingly, although the nomenclature of the resistance gene is usually from the abbreviation of the encoded product, such as the ampicillin resistance gene *bla* that encodes β-lactamase, for the convenience of description in this study, *AmpR*, *ChlR*, *GenR*, *KanR* are used to denote ampicillin, chloramphenicol, gentamicin and kanamycin resistance genes.

### 2.3. Assembly of Expression Vectors

**Figure 3:**
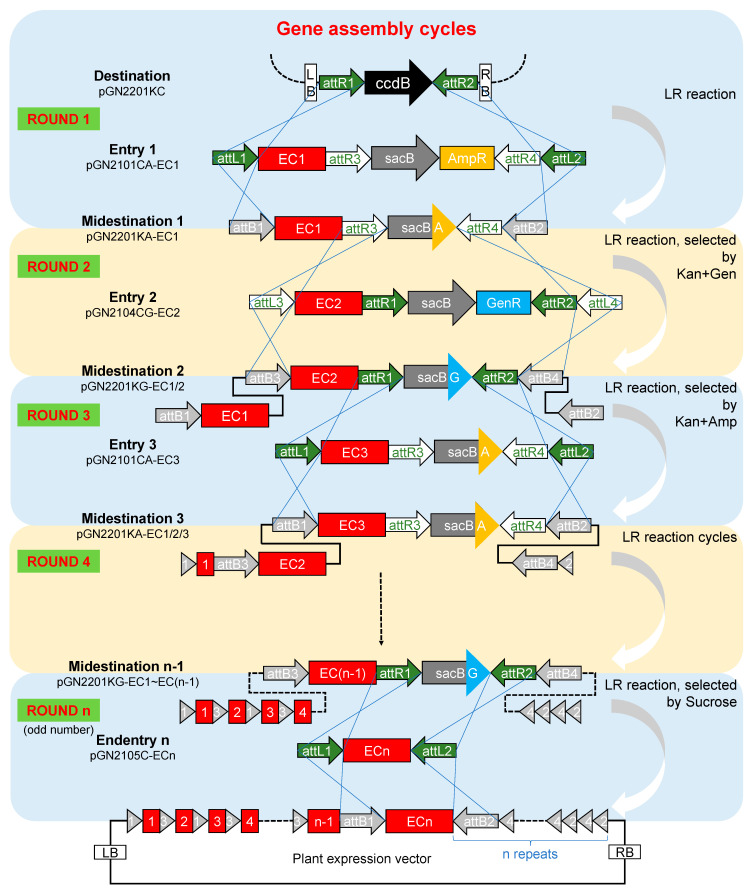
Repeated LR reaction-mediated gene stacking cycles. After preparing all the entry vectors that carry all the required transgenes, stacking cycles for integrating transgenes into a binary vector are ready to run. Each round of stacking is completed by LR recombination. In the first round, pGN2201KC was chosen as the destination vector, whose backbone contains *KanR*, and lethal *ccdB* linked with *ChlR* is located between *att*R1 and *att*R2. The entry vector for the round 1 LR reaction carries EC1, which is adjacent to *AmpR* and the sucrose sensitive gene *sacB*. The outmost *att*L1/*att*L2 can recombine with *att*R1/*att*R2 on the destination vector, and the *att*R3/*att*R4 integrated on the *ccdB*-free recombinant midestination is available for the next round of LR reaction. In the second round, EC2 and *att*R1/*att*R2 carried by entry vector 2 are integrated to the midestination of round 1, generating a new midestination screen out by kanamycin and gentamicin. Similar stacking processes are completed repeatedly, and different ECs are integrated. In odd rounds, target recombinants are selected by kanamycin and ampicillin, while in even rounds, target recombinants are selected by kanamycin and gentamicin. In the last step, stacking is completed by an endentry carrying the last EC, and the target recombinant is selected by sucrose.

Each round of stacking is completed via the LR reaction. In Figure 3, the basic GNS system is taken as an example to illustrate the specific stacking process of the expression vectors.

Stacking in round 1 requires the use of the corresponding entry vector for odd rounds, in which the backbone is a donor vector such as pGN2101CAK and the first EC is included; this vector may be referred to as pGN2101CA-EC1. pGN2101CA-EC1 and the destination vector pGN2201KC are then mixed for the LR reaction, and the target recombinant, midestination 1, is obtained by double screening using kanamycin and ampicillin.

Stacking in round 2 requires the use of the corresponding entry vector for even rounds, in which the backbone is a donor vector such as pGN2104CGK and the second EC is included; this vector may be referred to as pGN2104CG-EC2. pGN2104CG-EC2 and midestination 1 are then mixed for the LR reaction, and the target recombinant, midestination 2, is obtained by double screening using kanamycin and gentamicin.

By analogy, more cargos can be stacked on the binary vector backbone through repeated LR reactions.

All midestinations carry a pair of *att*R sites flanking linked positive and negative selection markers. Therefore, a midestination can be used as a destination vector for the next round of stacking but not as the final vector because prokaryotic selection markers in T-DNA are useless and may even be harmful in plant genetic engineering. The last round of the stacking reaction needs to be realized with an endentry containing only a pair of compatible *att*L sites. The target recombinant (the desired plant expression vector) will be obtained by reverse screening with sucrose.

### 2.4. Vector Library of the GNS System

Although the basic GNS system is sufficient for almost all scenarios of plant transgene stacking, we provide 64 vectors in total to improve the efficiency and flexibility of the GNS system (Table S1 and Supplementary Data S1).

**Figure 4:**
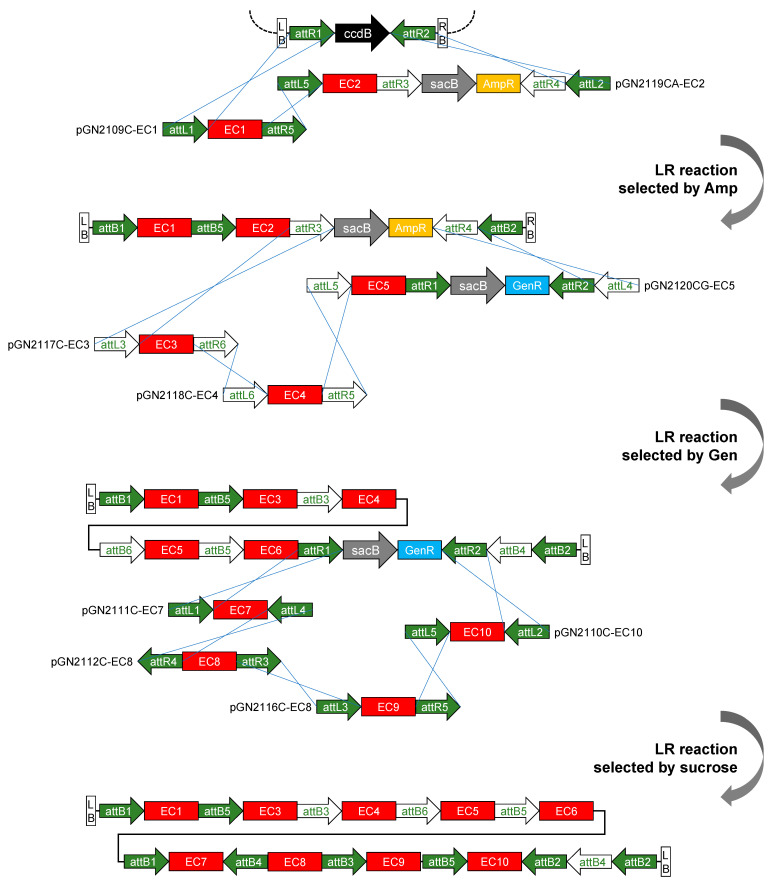
Multi-gene stacking in one round. Two entry vectors, based on the backbone of pGN2109CK and pGN2119CAK, can be assembled to a destination vector via 2-fragment multisite LR recombination. Then, three entry vectors based on the backbone of pGN2117CK, pGN2118CK and pGN2120CGK, can be assembled to the midestination subsequently via three-fragment multisite LR recombination. Then, four entry vectors based on the backbone of pGN2111CK, pGN2112CK, pGN2116CK and pGN2110CK can be assembled to the midestination subsequently via four-fragment multisite LR recombination.

A comprehensive library of GNS vectors can maximize the potential of the system. Briefly, the construction of the entry vector can be performed by either GG cloning or the BP reaction. Both of these cloning techniques have a well-established high efficiency. In addition, precloned element modules provided in the GNS library facilitate the GG assembly process. Different types of destination vectors that can meet specific needs, such as a single-copy insertion preference [22], marker-free transgene production [9] and a large-size T-DNA capacity [23] were also provided in the vector library. During the iterative LR reaction, a single cargo can be stacked similarly to other transgene stacking systems; besides that, two or three cargos can also be stacked via the GNS system. In the final reaction, up to four cargos can be stacked (Figure 4). The increase in the number of supported stacking cargos can meet the requirements for the assembly of superlarge ECs and even the construction of fusion proteins [24]. A detailed protocol for using the entire GNS system is provided in Supplementary File S1.

### 2.5. Construction of a Binary Vector, GMBPH, Containing Five Transcription Units

**Figure 5:**
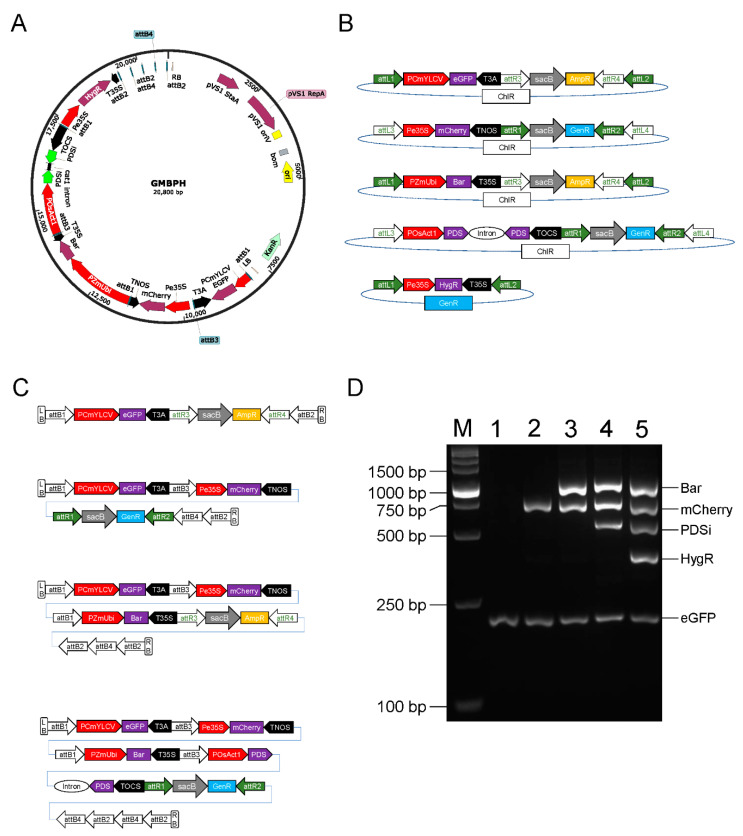
Construction of the binary vector GMBPH stacked five targets. (**A**) Map of GMBPH. PCmYLCV: *Cestrum* yellow leaf curling virus promoter; T3A: terminator of *RbcS* from *Pisum sativum*; Pe35S: enhanced 35S promoter from Cauliflower mosaic virus; TNOS: terminator of nopaline synthase from *Agrobacterium tumefaciens*; PZmUbi: *ubiquitin* promoter from *Zea mays;* T35S: terminator from Cauliflower mosaic virus; POsAct1: *actin1* promoter from *Oryza sativa*; PDSi: CDS of the *PDS* gene from *Oryza sativa*; cat1 intron: modified intron of catalase gene from castor bean; TOCS: terminator of octopine synthase gene from *A. tumefaciens*. (**B**) Map of five entry vectors for constructing GMBPH. From top to bottom are entry vector 1~4 and the endentry. (**C**) T-DNA region of four midestinations in the stacking process. From top to bottom are midestination 1~4. (**D**) PCR verification of all five binary vectors. M: DL5000 DNA marker; 1: pGN2201KA-eGFP; 2: pGN2201KG-EGFP-mCherry; 3: pGN2201KA-EGFP-mCherry-Bar; 4: pGN2201KG-EGFP-mCherry-Bar-PDSi; 5: the final expression vector GMBPH.

To test the effectiveness of the GNS system, we constructed the binary vector GMBPH, containing five ECs, including the green fluorescent protein gene *eGFP*, the red fluorescent protein gene *mCherry*, the glufosinate resistance gene *Bar*, a transcript unit for the knockdown of the rice phytoene desaturase-encoding gene *OsPDS* (LOC_Os03g08570), and the hygromycin resistance gene *HygR* (Figure 5A).

A total of five entry vectors were prepared for the construction of GMBPH. Among these vectors, entry vector 1, pGN2101CA-eGFP, consists of the backbone of the donor vector pGN2101CAK carrying an *eGFP* cassette including PCmYLCV (*Cestrum* yellow leaf curling virus promoter) [25] and T3A (the *RbcS* terminator of *Pisum sativum*) [26]. Entry vector 2, pGN2102CG-mCherry, consists of the backbone of the donor vector pGN2102CGK and carries an *mCherry* cassette including Pe35S (enhanced Cauliflower mosaic virus 35S promoter) [27] and TNOS (terminator of the nopaline synthase gene of *Agrobacterium tumefaciens*). Entry vector 3, pGN2101CA-Bar, carries the *Bar* gene driven by PZmUbi (*ubiquitin* promoter of *Zea mays*) and terminated by T35S (Cauliflower mosaic virus terminator). The EC in entry vector 4, pGN2102CG-PDSi, contains the POsAct1 (*actin1* promoter of *Oryza sativa*) and TOCS (terminator of the octopine synthase gene of *A. tumefaciens*) and transcribes double-stranded hairpin RNA (RNA interference, RNAi) to silence the endogenous rice gene *PDS* [28]. The last vector used for the final reaction, pDONR207-HygR, carries the *HygR* gene driven by Pe35S and terminated by T35S. (Figure 5B).

The vector pGN2201KC was chosen as the destination vector for stacking multiple genes, and four midestinations and one final expression vector were obtained. Midestination 1, pGN2201KA-eGFP, expressing eGFP, was obtained in the first round of stacking and is resistant to kanamycin and ampicillin. Midestination 2, pGN2201KG-EGFP-mCherry, contains two ECs of eGFP and mCherry and is resistant to kanamycin and gentamicin. Midestination 3, pGN2201KA-EGFP-mCherry-Bar, contains three ECs of *eGFP*, *mCherry* and *Bar* and is resistant to kanamycin and ampicillin. Midestination 4, pGN2201KG-EGFP-mCherry-Bar-PDSi, contains four ECs of *eGFP*, *mCherry*, *Bar* and *PDSi* and is resistant to kanamycin and gentamicin. The final vector obtained in the last round of stacking, pGN2201K-EGFP-mCherry-Bar-PDSi-HygR (referred to as GMBPH), contains all five ECs of *eGFP*, *mCherry*, *Bar*, *PDSi* and *HygR* and only contains a kanamycin resistance gene outside of the T-DNA border (Figure 5C).

PCR identification, Sanger sequencing and next-generation sequencing (NGS) proved the successful construction of GMBPH (Figure 5D and Supplementary Data S2).

### 2.6. Genotype and Phenotype Identification of GMBPH Transformed Rice

**Figure 6:**
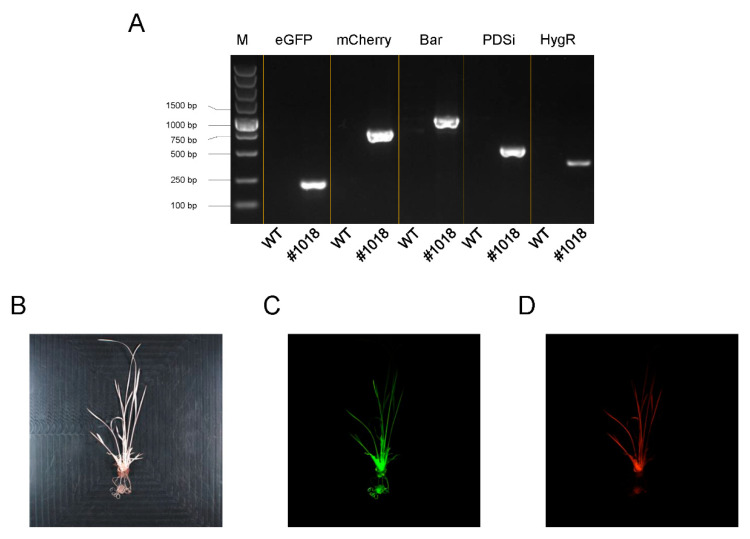
Investigation of GMBPH transformed rice. (**A**) PCR identification of transgenic rice. Primer pairs for detecting corresponding cargos of GMBPH were marked on the top. PCR templates, wild type Nipponbare and transgenic line 1018 were marked under the bottom. (**B**) Photo of transgenic line 1018. (**C**) Detection of GFP fluorescence. (**D**) Detection of mCherry fluorescence.

More than 10 independent transgenic lines were obtained, and one line denoted as #1018 was chosen for further investigation. PCR identification proved the existence of all five units of GMBPH in the transgenic line #1018 (Figure 6A). As expected, T0 plants grew well on medium containing hygromycin and glufosinate, indicating the expression of *HygR* and *Bar*. The majority of T0 plants were albino, as shown in the phenotype of #1018 (Figure 6B), indicating the expression of the RNAi cassette of *PDS*. Green and red fluorescence was observed in #1018 (Figure 6C,D), indicating the expression of *eGFP* and *mCherry*.

## 3. Discussion

In developing a transgene stacking system, it first needs to be determined whether the stacking process will be implemented in vivo or in vitro. Most reported systems choose in vivo stacking, in which the tool enzymes are expressed in host cells, saving commercial purchase costs. However, tool enzymes represented by site-specific recombinases are not naturally present in hosts; that requires that the host cells be genetically modified before stacking. Although there are some exceptions, for example, jStack [10] uses a yeast endogenous homologous recombination mechanism, this strategy is risky for constructing complex vectors with repetitive sequences [8]. Moreover, in vivo stacking takes longer than in vitro stacking. Considering all of these factors, in vitro stacking was chosen for the GNS system.

A transgene stacking system needs to solve four problems. First, the replication ability of large plasmids is limited in *E. coli* and *Agrobacterium*. Second, conventional recombinant plasmid construction involves linearization and recircularization, and recovery and circularization are difficult for large plasmids. Third, in designing the stacking mechanism, the intermediate vectors must retain the ability to accept the next round of stacking, and at the same time, stacking before and after must not conflict. Fourth, bacterial transformation using large plasmids is inefficient.

It has been reported that the application of a phage replicon instead of the commonly used pBR322 replicon can guarantee the efficient replication of large plasmids in bacteria [29]. In this study, destination vectors with the TAC backbone in the TGSII system were provided, which included the phage P1 replicon and the lytic P1 replicon [9].

When in vitro molecular cloning is performed, the difficulty of recovering and circularizing linear DNA molecules restricts commonly used methods through sticky-end induction by endonucleases or exonucleases, followed by ligation. In addition, methods using restriction endonucleases that recognize a few base pairs are often unavailable for large fragments. Site-specific DNA recombinases, mainly divided into tyrosine recombinases such as λ Int (LR Clonase) and Cre and serine recombinases such as ϕC31 integrase, act as topoisomerases with instantaneous cleavage and ligation abilities, providing an alternative option for generating recombinant DNA molecules without plasmid linearization and recircularization [30]. In addition, site-specific recombination with longer recognition sequences will eliminate the feature dependence of target DNA fragments for molecular cloning. However, the mechanism of site-specific recombinases is inconsistent with the requirement for stacking. If the recombination site is lost in the stacked intermediate, stacking will be unavailable in the next round. If the recombination site is retained, the recombinase expected to transfer new cargo to an entry vector may misrecognize previously integrated cargos in the intermediate. In the TGSII system, the Cre enzyme is used and is paired with an ingeniously designed *lox*P site combination to ensure that recombination in different rounds will not conflict [9]. In this study, the mature commercial in vitro recombinase LR Clonase was selected. Unlike the Cre enzyme, whose application is focused on solving the conflict between recombination, the *att*B sites generated in the recombinant molecule in the LR reaction will no longer be recognized by LR Clonase. Thus, no conflict will occur when using the LR enzyme, but the next cargo cannot be stacked unless new *att*R sites are added. Nevertheless, the LR enzyme can specifically recognize recombination sites with different types of mutations. If a pair of *att*R sites linked with a cargo is inserted into the incompatible *att*L sites of an entry vector, it will not only ensure correct recombination but will also ensure the capacity of the intermediates to receive another LR reaction. This strategy has been described in a previous report [7]. The use of LR Clonase as the enzyme tool for stacking may also be readily compatible with the marker-free transgene produced by the Cre/*lox*P system, and corresponding element modules are provided in the GNS plasmids (Table S1).

The electroporation transformation method combined with special competent strains, such as DH10B and stbl4, can overcome the limitation of transforming *E. coli* with complex plasmids, such as plasmids that have a large size or contain excessive repetitive sequences [31,32]. In addition to the target recombinants, the obtained colonies often contain nontarget colonies, including empty vector transformants and miscellaneous bacteria. Therefore, a complete cloning process requires strategies including positive selection and negative selection to identify target colonies. Positive selection efficiency is high, but the target recombinant has to carry a selection marker other than the target fragment, while scarless negative screening requires a specific host genotype, and the efficiency is lower due to possible counterselection escape [33,34]. The commonly used negative selection marker for the Gateway reaction is *ccdB*, which is a nonconditionally lethal gene [19]. The constitutive expression of *ccdB* in common strains is lethal except in a few ccdB-tolerant strains, such as DB3.1 [35], and is therefore obviously not suitable for large-plasmid transformation. The expression of *sacB* [20] is generally not harmful to hosts, except for the addition of exogenous sucrose. Therefore, in the GNS system, the linkage of antibiotic genes and the sucrose-sensitive gene *sacB* has been proposed as a solution. Stacking in odd rounds and even rounds is positively selected using ampicillin and gentamicin, respectively, resulting in high efficiency. In the final reaction, sucrose is added to the medium to negatively select *sacB*-free recombinants. Therefore, the selection strategy in the GNS system takes full advantage of the high efficiency of positive selection; at the same time, no unwanted antibiotic markers will be introduced in the final vector.

In the GNS system, all midestinations include a combination of *att*R sites, among which the *att*R1/*att*R2 combination is employed in odd rounds to accept the stack of cargo carried by the next entry vector, and *att*R3/*att*R4 is used in even rounds. Therefore, each midestination is capable of assembling multiple cargos via a multisite LR reaction [15]. Of course, the entry vectors and midestinations for non-final stacking carry at least four *att*L/*att*R sites, and the number of cargos stacked by multi-site LR reactions cannot reach the upper limit (i.e., the assembly of four cargos at the same time). Nevertheless, the assembly of three cargos at one time is possible through the rational redesign of recombination sites. The introduction of multisite recombination would further expand the stacking ability and freedom of the GNS system.

Overall, in addition to the advantages of the MultiRound Gateway system [7], such as faster completion due to in vitro reactions, the GNS system has some unique advantages as described below. First, the introduction of a host-independent and controllable negative selection marker allows freedom of receptor choice. *E. coli* strains with special characteristics such as DH10B and stbl4 can be used throughout the process, guaranteeing the easy transformation of complex plasmids. Second, the introduction of the TAC backbone with the P1 replicon and lytic P1 replicon can guarantee the correct and highly efficient replication of plasmids longer than 100 kb [9,23,36]. Third, entry vectors can be assembled via GG cloning, thus suggesting a modular solution. Fourth, entry vectors can be assembled via the BP reaction, thus guaranteeing the highly efficient assembly of large cargos such as genomic DNA fragments longer than 10 kb. Fifth, positive selection is adopted in intermediate stacking, thus decreasing the false-positive rate, and negative selection is adopted in the last step, so that no unwanted antibiotic genes remain in the expression clones. This strategy combines the advantages of the high efficiency of positive selection and the scarlessness of the negative selection. Sixth, up to three cargos can be stacked in one round, and up to four cargos can be stacked in the last reaction, further improving the flexibility and efficiency of the system.

## 4. Materials and Methods

### 4.1. Plant, Bacterial and Vector Material

*Japonica* rice cultivar Nipponbare (*Oryza sativa* sp. *geng* cv. Nipponbare) was used for transformation in this study. Bacterial competent cells included the *Agrobacterium tumefaciens* strain AGL1 (ZC144) and *E. coli* strain DH5α (ZC101), DH10B (ZC112) and DB3.1 (ZC109) was purchased from ZOMANBIO (Beijing, China). All the donor vectors were based on pGGAselect backbone, which was provided in the Golden Gate Assembly Kit (BsaI-HF^®^v2) (NEB, E1601). The destination vectors were based on pCambia1300 backbone and TAC backbone. TAC vector backbone was obtained from TGSII system [9], which was kindly provided by Prof. Yao-Guang Liu (South China Agricultural University).

### 4.2. Construction of GNS Vector Library

Necessary elements were PCR amplified (2×Phanta Max Mix, Vazyme, P525, Nanjing, China) using available templates or synthesized chemically (Sangon Biotech, Shanghai, China). The plasmids were assembled through different methods, including BP reaction (11789020, Invitrogen, Carlsbad, CA, USA), TOPO cloning (CL071 and CL119, Biomed, Beijing, China), Gibson cloning (Trelief SoSoo Cloning Kit, TsingKe, Beijing, China) and GG Cloning using Type IIS restriction endonuclease *Bsa*I (R3733, NEB, Ipswich, MA, USA), *BsmB*I (R0739, NEB, Ipswich, MA, USA) or *Aar*I (ER1581, Thermo Scientific, Waltham, MA, USA) as well as T4 ligase (M0202, NEB, Ipswich, MA, USA). All the plasmids in the GNS vector library are available from the corresponding authors upon request, and will be deposited on Addgene. 

### 4.3. Construction of the Binary Vector GMBPH

In constructing entry vectors, the Type IIS restriction endonuclease *Bsa*I (R3733, NEB, Ipswich, MA, USA), T4 ligase (M0202, NEB, Ipswich, MA, USA)-mediated GG cloning and Gateway™ BP Clonase™ II Enzyme mix (11789020, Invitrogen, Carlsbad, CA, USA)-mediated BP recombination were used.

In entry 1 (pGN2101CA-eGFP), the pGN2101CAK backbone and 3 element modules, including pGN2301G, pGN2441S and pGN2502A, were used to assemble the PCmYLCV:eGFP:T3A expression cassette by GG cloning. In entry 2 (pGN2102CG-mCherry), the pGN2102CGK backbone and 3 element modules, including pGN2302G, pGN2442S and pGN2506A, were used to assemble the Pe35S:mCherry:TNOS expression cassette by GG cloning. In entry 3 (pGN2101CA-Bar), the pGN2101CAK backbone and 3 element modules, including pGN2312G, pGN2431S and pGN2501G, are used to assemble the PZmUbi:Bar:T35S expression cassette by GG cloning. In entry 4 (pGN2102CG-PDSi), the pGN2102CGK backbone, 2 element modules (pGN2307G and pGN2507A) and precloned inserts including Os*PDS* and intron sequences were used to assemble the POsAct1:PDSi:TOCS expression cassette by GG cloning. Entry 5 (the endentry, pDONR207-HygR) was constructed by the BP reaction using the donor vector pDONR207 and the *att*B-flanked amplicon of Pe35S:HygR:T35S.

For the stacking reaction, Gateway™ LR Clonase™ II Enzyme mix (Invitrogen, 11791020)-mediated LR recombination was used.

In round 1 of stacking, pGN2201KC and entry 1 were mixed to perform the LR reaction. The liquid product was then transformed into *E. coli* DH10B competent cells, which were subsequently selected by kanamycin plus ampicillin treatment to obtain midestination 1, pGN2201KA-eGFP. In round 2 of stacking, midestination 1 and entry 2 were mixed to perform the LR reaction, after which *E. coli* DH10B was transformed under kanamycin plus gentamicin selection pressure to obtain midestination 2, pGN2201KG-EGFP-mCherry. In round 3 of stacking, midestination 2 and entry 3 were mixed to perform the LR reaction, and *E. coli* DH10B was then transformed under kanamycin plus ampicillin selection pressure to obtain midestination 3, pGN2201KA-EGFP-mCherry-Bar. In round 4 of stacking, midestination 3 and entry 4 were mixed to perform the LR reaction, and *E. coli* DH10B was then transformed under kanamycin plus gentamicin selection pressure to obtain midestination 4, pGN2201KG-EGFP-mCherry-Bar-PDSi. In round 5 of stacking, which was the last reaction, midestination 4 was mixed with the endentry to perform the LR reaction, and *E. coli* DH10B was subsequently transformed under sucrose selection pressure to obtain the final expression clone, GMBPH.

A set of 5 primer pairs were used for PCR detection of the 5 cargos in GMBPH. PCR reactions were performed independently, and the products were mixed equally for detection via 1% agarose gel electrophoresis. Primers are provided in Table S2. Sanger sequencing and NGS sequencing of GMBPH were completed by TsingKe commercially.

A specific and detailed protocol is provided in Supplementary File S1.

The vector GMBPH is available upon request.

### 4.4. Rice Transformation

Mature dehusked rice seeds were sterilized and inoculated on MS medium containing 4 mg/L 2,4-D for calli induction. Actively growing calli were collected for subculture for 1 week. The binary vector GMBPH was introduced into *A. tumefaciens* strain AGL1. Good calli were selected and immersed in *A. tumefaciens* suspension (with 100 μM acetosyringone) for 30 min and then cultured on NB medium (with 100 μM acetosyringone) at 22 ℃ for 3 d. Then, the calli were rinsed and transferred onto NB medium (with 50 mg/L hygromycin) for 2-week selection. Resistant calli were transferred onto fresh screening medium for another selection stage. Pink calli (red fluorescence protein expressed) showing hygromycin resistance after 2 rounds of selection were moved onto NB medium (with 3.6 mg/L 6-BA and 0.6 mg/L NAA) for regeneration. After more than 3 weeks, regenerated seedlings were transferred onto 1/2 MS medium (with 6 mg/L glufosinate) for 2 weeks. Albino seedlings with *OsPDS* interfered strongly, were incapable of photosynthesis, and could only survive on mediums, while pale green plants could be transferred into soil.

### 4.5. Identification of GMBPH Transformed Rice

DNA was extracted from the leaves of GMBPH transformed seedlings (T0) using Hi-DNAsecure Plant Kit (DP350, TIANGEN, Beijing, China). PCR detection was performed with the identical primers used in detecting GMBPH vector. The green and red fluorescence were detected using an imaging system (FUSION FX SPECTRA, VILBER, Collégien, France).

## Figures and Tables

**Table 1:** Vectors in basic GNS system.

Vector Name	Vector Category	Components	Backbone Resistance	Additional Resistance	Usage
pGN2101CAK	Donor	*att*L1 = *KanR* + *ccdB* = *att*R3-*sacB* + *AmpR*-*att*R4-*att*L2 ^1^	Chl	Amp and Kan	Creating an entry vector for odd-round stacking through GG cloning
pGN2102CGK	Donor	*att*L3 = *KanR* + *ccdB* = *att*R1-*sacB* + *GenR*-*att*R2-*att*L4	Chl	Gen and Kan	Creating an entry vector for even-round stacking through GG cloning
pGN2103CAK	Donor	*att*P1-*KanR* + *ccdB*-*att*P3r-*sacB* + *AmpR*-*att*R4-*att*L2	Chl	Amp and Kan	Creating an entry vector for odd-round stacking through BP reaction
pGN2104CGK	Donor	*att*P3-*KanR* + *ccdB*-*att*P1r-*sacB* + *GenR*-*att*R2-*att*L4	Chl	Gen and Kan	Creating an entry vector for odd-round stacking through BP reaction
pGN2105CK ^2^	Donor	*att*L1 = *KanR* + *ccdB* = *att*L2	Chl	Kan	Creating an endentry for odd-round stacking through GG cloning
pGN2106CK ^3^	Donor	*att*L3 = *KanR* + *ccdB* = *att*L4	Chl	Kan	Creating an endentry for even-round stacking through GG cloning
pGN2201KC	Destination	LB = *att*R1-*ChlR* + *ccdB*-*att*R2 = RB	Kan	Chl	Binary vector for accepting cargo on the first entry vector

^1^ An equal sign represents a recognition site of *Bsa*I restriction enzyme. ^2^ Endentries with *att*L1/*att*L2 combination can also be constructed using other cloning methods such as TOPO cloning, traditional restriction enzyme and ligase method, and BP recombination [21] with commercially available vectors. The GNS system also provides vector options for constructing *att*L1/*att*L2 endentries via BP recombination (Table S1). ^3^ The GNS system also provides vector options for constructing *att*L3/*att*L4 endentries via BP recombination (Table S1).

## Data Availability

The data presented in this study are available in this article and the Supplementary Material.

## Supplementary Materials

The following are available online at https://www.mdpi.com/article/10.3390/plants11040488/s1, Supplementary Data S1: Plasmid map of all the vectors used in this study, Supplementary Data S2: The sequencing data of the GMBPH plasmid, Supplementary File S1: Standard protocol for constructing multigene binary vectors via GNS system, Table S1: Detailed plasmid information of the entire GNS system, Table S2: Primers used in this study.
