# Supplementary material for: An Efficient Modular Gateway Recombinase-Based Gene Stacking System for Generating Multi-Trait Transgenic Plants
Source: Plants (Basel). 2022 Feb 11;11(4):488. doi: 10.3390/plants11040488 (PMC8879548; doi:10.3390/plants11040488)
Supplement: Supplementary file 1 [file plants-11-00488-s001.zip › Supplementary file S1.pdf]

Standard protocol for constructing multigene binary vectors via GNS system

1. Construction of entry vectors via Golden Gate cloning or BP recombination

Single cargo stacking only needs the application of vectors of the basic GNS system (Table 1). The specific combination of donor vectors has been mentioned in the text. Multiple cargo stacking needs more complex combinations of donor vectors as described in the table below. Note that donor vectors carrying *ccdB* lethal gene need to use *Escherichia coli* strain DB3.1 for propagation.

A 15-μL GG cloning was set up as follows: 1 μL of donor vector (~50 ng/μL), precloned inserts or amplicons, 1.5 μL of CutSmart Buffer, 1.5 μL of T4 Buffer, 1 μL of *Bsa*I-HF v2, 1 μL of T4 ligase and ddH_2O to make up. The molar ratio of inserts and the backbone ranged from 2 to 5 and the NEBiocalculator® Tool (https://nebiocalculator.neb.com/) can be used for molar calculations. The reagents were purchased from NEB, with catalog number mentioned in materials and methods. Golden Gate assembly procedures were as follows: 37°C for 15 min; 37°C for 5 min, 10°C for 5 min, 20°C for 5 min, 15 cycles； 37°C for 15 min and 80°C for 20 min. If the product is not used to transform *E. coli* immediately, it needs to be transferred to -20°C.

Cargos need to introduce different types of *att*B sites by PCR through primers with adapters (Wall et al., 2014). A 5 μL BP reaction was set up as follows: donor vector (~100 ng), *att*B-flanked cargo (~200 ng), 1 μL of BP recombinase Mix and ddH_2O to make up. The reagents were purchased from Invitrogen, with catalog number mentioned in materials and methods. After incubating at 25°C for at least 16 h, the recombinant products are used to transform *E. coli* or stored in -20°C.

After transforming *E. coli* DH5α competent cells, chloramphenicol or gentamycin is used for positive selection, depending on the antibiotic gene of the donor backbones.

2. Construction of midestinations via LR recombination

Any binary vector with *att*R1/*att*R2 sites can be used as a destination vector. In the GNS system, diversified destination vectors are provided, all of which are specifically domesticated to remove *Bsa*I and *BsmB*I recognition sites. Among them, pGN2201KC~pGN2204KC are based on pCambia1300 backbone and pGN2211KC~pGN2212KC are based on TAC backbone (Liu et al., 1999). The pGN2204KC are specifically designed for obtaining marker-free and single-copy transgenic plants (Srivastava et al., 1999; Zhu et al., 2017). Binary vectors obtained by the GNS system will have a repetitive sequence on the left side of right border. The vector stability may not be affected because the repetitive sequence is short, and the expression may not be affected because the repetitive sequence is located outside all the cargos. But the identification of T-DNA flanking sequence on transgenic plants will be more complex. To solve this problem fundamentally, a recognition site for homing endonuclease *I-Ceu*I was added between attR2 and RB of pGN2211KC. Endentry based on pGN2133CK or pGN2134CK also carries an *I-Ceu*I recognition site behind cargos, thus providing a possibility to remove the repetitive sequence on the expression vector. Similarly, the combination of destination vector pGN2212KC and

endentry based on pGN2135CK or pGN2136CK backbone can remove the repetitive sequence on the expression vector by CRE-mediated fragment deletion.

A 10 μL LR reaction was set up as follows: entry vectors (10 fmoles each), destination vector (20 fmoles), 2 μL of LR recombinase Mix and ddH_2O to make up. The reagents were purchased from Invitrogen. Note that Gateway™ LR Clonase™ II Enzyme mix (11791020) is applicable for stacking no more than 2 cargos. LR Clonase™ II Plus enzyme (12538120) is recommended to stack 3 or 4 cargos. After incubating at 25°C for at least 16 h, the recombinant products are used to transform *E. coli* or stored in -20°C.

After transforming *E. coli* DH10B competent cells, double antibiotics are used for positive selection. The *KanR* on the destination backbone confers kanamycin resistance while the *AmpR* or *GenR* linked with *SacB* confer ampicillin or gentamycin resistance. Typically, kanamycin and ampicillin are used in odd round while kanamycin and gentamicin are used in even round. Note that for TAC-based destination vectors with the P1 lytic replicon, the LB medium contains 0.5 mM IPTG.

3. Construction of the expression vector via LR recombination

An endentry is used for the final LR recombination and the reaction was set up as mentioned before.

After transforming *E. coli* DH10B competent cells, kanamycin and sucrose are used for positive and negative selection.

4. Follow-up experiments

The constructed expression vectors can be used for plant transformation using *Agrobacterium*-mediated method or bombardment particle method.

REFERENCE

Liu, Y.-G., Shirano, Y., Fukaki, H., Yanai, Y., Tasaka, M., Tabata, S. and Shibata, D. (1999) Complementation of plant mutants with large genomic DNA fragments by a transformation-competent artificial chromosome vector accelerates positional cloning. *Proceedings of the National Academy of Sciences* **96**, 6535-6540.

Srivastava, V., Anderson, O.D. and Ow, D.W. (1999) Single-copy transgenic wheat generated through the resolution of complex integration patterns. *Proceedings of the National Academy of Sciences* **96**, 11117-11121.

Wall, V.E., Garvey, L.A., Mehalko, J.L., Procter, L.V. and Esposito, D. (2014) Combinatorial Assembly of Clone Libraries Using Site-Specific Recombination. In: *DNA Cloning and Assembly Methods* (Valla, S. and Lale, R. eds), pp. 193-208. Totowa, NJ: Humana Press.

Zhu, Q., Yu, S., Zeng, D., Liu, H., Wang, H., Yang, Z., Xie, X., Shen, R., Tan, J., Li, H., Zhao, X., Zhang, Q., Chen, Y., Guo, J., Chen, L. and Liu, Y.-G. (2017) Development of “Purple Endosperm Rice” by engineering anthocyanin biosynthesis in the endosperm with a high-efficiency transgene stacking system. *Mol Plant* **10**, 918-929.

Assembly method for constructing an entry vector	Donor vector backbones	Purpose
GG assembly	pGN2101 CAK	1-cargo stacking in odd-round for constructing a midestination
BP recombination	pGN2103 CAK	
GG assembly	pGN2109 CK, pGN2119 CAK	2-cargo stacking in odd-round for constructing a midestination
BP recombination	pGN2122 CK, pGN2131 CAK	
GG assembly	pGN2115 CK, pGN2118 CK, pGN2119 CAK	3-cargo stacking in odd-round for constructing a midestination
BP recombination	pGN2127 CK, pGN2130 CK, pGN2131 CAK	
GG assembly	pGN2105 CK	1-cargo stacking in odd-round for constructing the expression vector
	pGN2133 CK	
	pGN2135 CK	
BP recombination	pGN3101 GC	
GG assembly	pGN2109 CK, pGN2110 CK	2-cargo stacking in odd-round for constructing the expression vector
BP recombination	pGN2122 CK, pGN2124 CK	
GG assembly	pGN2115 CK, pGN2118 CK, pGN2110 CK	3-cargo stacking in odd-round for constructing the expression vector
BP recombination	pGN2127 CK, pGN2130 CK, pGN2124 CK	
GG assembly	pGN2111 CK, pGN2112 CK, pGN2116 CK, pGN2110 CK	4-cargo stacking in odd-round for constructing the expression vector

BP recombination	pGN2121<br>CK	pGN2123<br>CK	pGN2128<br>CK	pGN2124<br>CK	
GG assembly	pGN2102<br>CGK				1-cargo stacking in even-round for constructing a midestination
BP recombination	pGN2104<br>CGK				
GG assembly	pGN2116<br>CK	pGN2120<br>CGK			2-cargo stacking in even-round for constructing a midestination
BP recombination	pGN2128<br>CK	pGN2132<br>CGK			
GG assembly	pGN2117<br>CK	pGN2118<br>CK	pGN2120<br>CGK		3-cargo stacking in even-round for constructing a midestination
BP recombination	pGN2129<br>CK	pGN2130<br>CK	pGN2132<br>CGK		
GG assembly	pGN2106<br>CK				1-cargo stacking in even-round for constructing the expression vector
	pGN2134<br>CK				
	pGN2136<br>CK				
BP recombination	pGN3102<br>GC				
GG assembly	pGN2116<br>CK	pGN2114<br>CK			2-cargo stacking in even-round for constructing the expression vector
BP recombination	pGN2128<br>CK	pGN2125<br>CK			
GG assembly	pGN2117<br>CK	pGN2118<br>CK	pGN2114<br>CK		3-cargo stacking in even-round for constructing the expression vector
BP recombination	pGN2129<br>CK	pGN2130<br>CK	pGN2125<br>CK		
